# Pityriasis Versicolor in a Southern Ground Hornbill (*Bucorvus leadbeateri*)

**DOI:** 10.1155/crve/5261490

**Published:** 2026-02-23

**Authors:** Gonçalo N. Marques, Miguel Lourenço, Miriam Leal, Nuno Urbani, Maria Conceição Peleteiro

**Affiliations:** ^1^ Veterinary Department, Zoomarine Portugal, Guia, Portugal; ^2^ MARE-Marine and Environmental Sciences Centre/ARNET-Aquatic Sciences Network, ISPA-Instituto Universitário de Ciências Psicológicas, Sociais e da Vida, Lisbon, Portugal; ^3^ Pathology Department, URANOLABPT, Avenida Pedro Álvares Cabral, Sintra, Portugal

## Abstract

A 20‐year‐old southern ground hornbill (*Bucorvus leadbeateri*) developed hyperpigmented macules on its gular pouch, resembling the lesions typically seen in humans with pityriasis versicolor. Cytologic examination revealed over 100 budding yeasts per oil immersion field. Fungal culture showed rare growth of smooth, cream‐colored yeast colonies, identified by PCR as *Malassezia slooffiae*. Histopathological analysis showed lymphocytic perivascular dermatitis. Periodic acid–Schiff staining revealed rare forms of yeasts between the layers of the stratum corneum. This clinical report provides further insights into the role of *Malassezia* spp. in the avian skin microbiome. To the authors′ knowledge, this is the first report of *Malassezia* sp. as an agent of pityriasis versicolor in birds.

## 1. Introduction

The southern ground hornbill (*Bucorvus leadbeateri*) is listed as “vulnerable” on the IUCN Red List of Threatened Species, although it is classified as “endangered” in South Africa and Namibia [1]. Populations in South Africa continue to decline toward a “critically endangered” status [1]. While several in situ measures are being explored, zoo populations for this species are increasingly important as a safety net [2]. As of January 2025, Species360 reports a population of over 397 individuals across 167 institutions.

*Malassezia* spp. are lipophilic yeasts that have been described as part of the skin and mucosal microbiota of some mammals and may act as opportunistic pathogens [3]. In domestic mammals, *Malassezia* spp. are frequently implicated as secondary agents of otitis externa and dermatitis [4, 5]. Birds, however, have been less frequently studied as carriers of *Malassezia* spp., though these fungi have been isolated from different healthy and diseased sites (e.g., beak, oral cavity, oropharynx, skin, feathers, comb, and feces) among various bird species [3, 4]. *Malassezia* species isolated from birds include *Malassezia pachydermatis*, *Malassezia furfur*, *Malassezia sympodialis*, *Malassezia brasiliensis* sp. nov., *Malassezia psittaci* sp. nov., *Malassezia globosa*, *Malassezia restricta*, and *Malassezia slooffiae* [3, 4]. These yeasts have been reported as opportunistic pathogens in cases of avian dermatitis, feather‐destructive behavior, systemic isosporosis, macrorhabdosis, invasive candidiasis, and avian pox [6, 7]. Brasão et al. reported a small, white, rounded lesion at the edge of the right eye of an *Aratinga leucophthalma*, from which *Malassezia* sp. was isolated [8]. Moreover, Breuer‐Strosberg et al. reported the isolation of *M. pachydermatis* from a 2‐year‐old female scarlet macaw (*Ara macao*) that presented with weight loss, weakness, and voice changes. The pharynx and larynx were described as markedly reddened and swollen [9]. To the authors′ knowledge, there have been no previous reports on the role of *Malassezia* spp. as agents of skin depigmentation in birds.

## 2. Case Presentation

**Figure 1:**
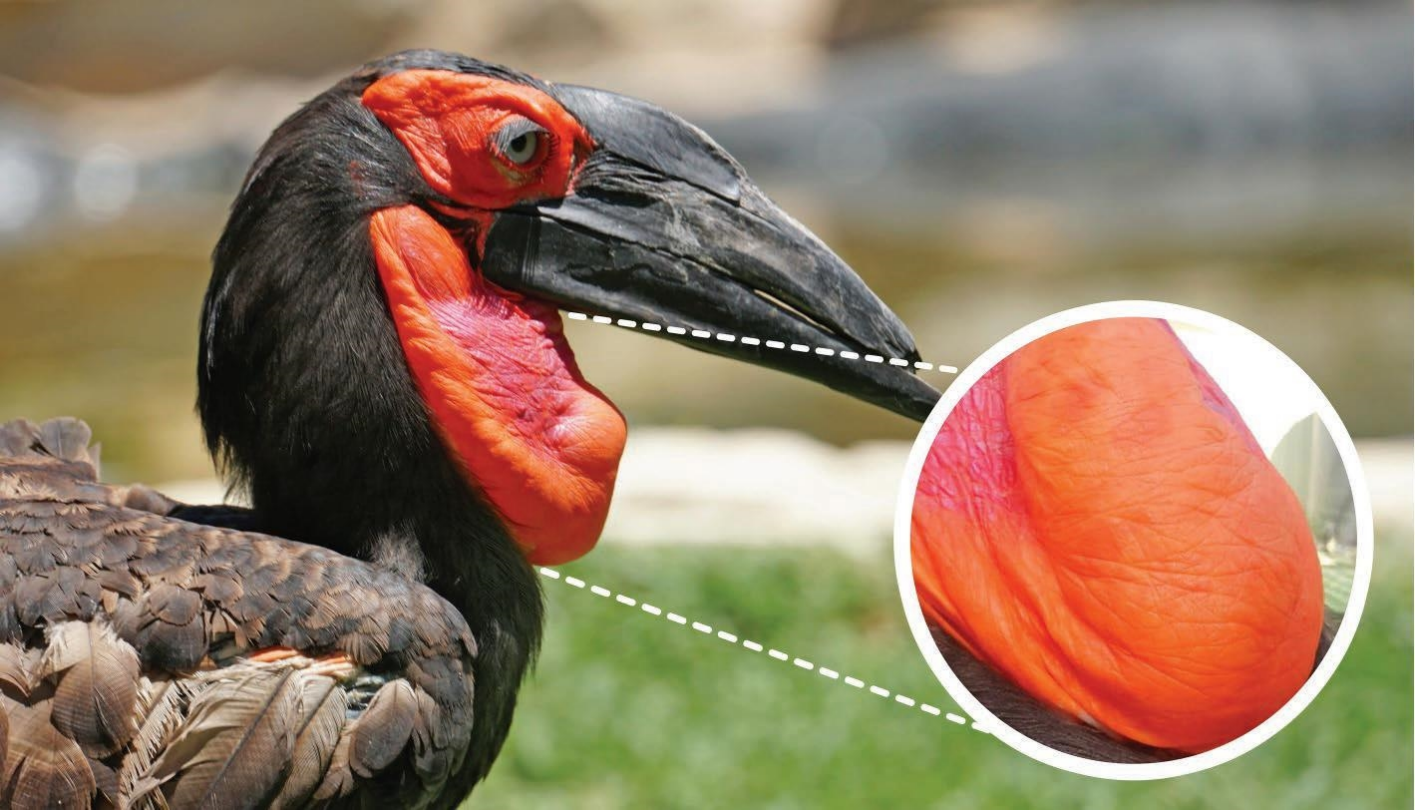
Hyperpigmented macules in the gular pouch of a southern ground hornbill (*Bucorvus leadbeateri*).

A 20‐year‐old male southern ground hornbill housed under professional care at Zoomarine developed several roundish, hyperpigmented macules, up to 10 mm in diameter, in its gular pouch (Day 0) (Figure 1). The lesions were flat and nonscaly, with no signs of scabs, pustules, or vesicles, and did not cause pruritus. Physical examination and bloodwork showed no alterations. The bird′s medical history was overall unremarkable, with no previous reports of skin disease.

**Figure 2:**
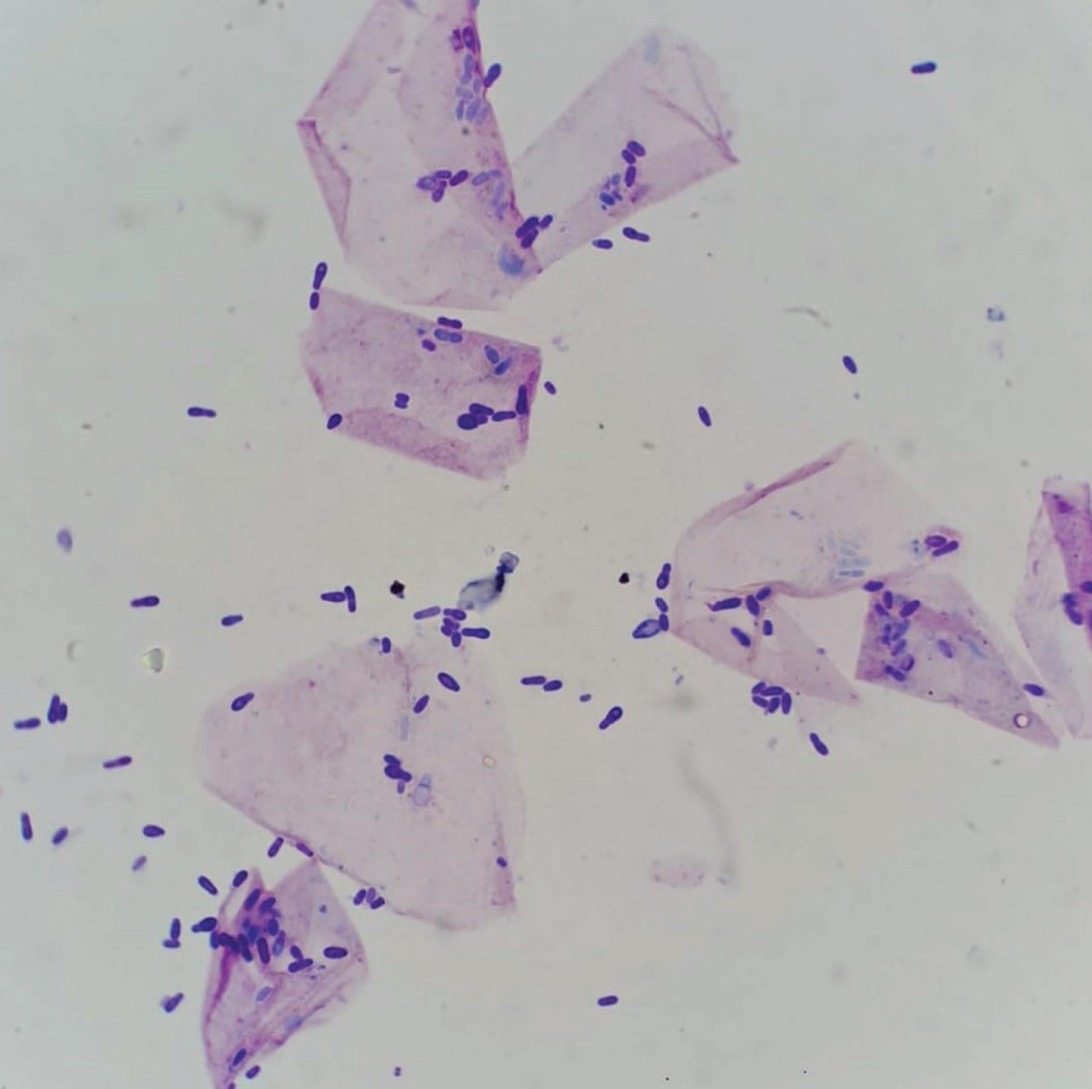
Impression smear of the hyperpigmented macules—budding yeasts morphologically coincident with *Malassezia* sp. (Diff‐Quik, ×1000).

For microscopic examination of cutaneous preparations, samples were collected through impression smears, in which the glass slide was pressed repeatedly on the lesion, or by pressing a strip of clear tape on the skin. In both cases, Diff‐Quik staining was used. Slides were then observed under the microscope (×40–×1000 with oil immersion). Cytological examination revealed more than 100 budding yeasts/oil immersion field (OIF), morphologically coincident with *Malassezia* sp. (Day 30) (Figure 2). Bacteria were scarcely observed. No inflammatory cells were seen. Yeasts were rarely detected in the cytological evaluation of other healthy skin areas—zero yeasts/OIF in the chest area and up to eight yeasts/OIF in the periocular skin. Lesion monitoring was conducted through direct observation and cytological evaluation. Table 1 summarizes cytology results throughout the clinical case.

**Table 1:** Cytological evaluation of the gular pouch skin throughout the clinical case.

Date (day/month/year)	Day of the clinical case	Number of *Malassezia* sp. forms/oil immersion field	Clinical status (macules)
10/04/2024	30	> 100	Present
05/05/2024	55	10	Present
12/05/2024	62	0–1	Resolving
20/07/2024	131	1	Almost resolved
25/08/2024	167	0–1	Almost resolved
08/09/2024	181	0–1	Almost resolved
14/01/2025	309	20	Recurred

Skin fungal in‐house cultures were performed on Sabouraud Dextrose Chloramphenicol Agar at both 25°C and 37°C for 5–7 days, with and without supplementation of a thin film of olive oil above the solid medium. Rare growth of smooth and cream‐colored colonies was observed in the cultures at 37°C, with oil supplementation (Figure 3a). Microscopic morphology was consistent with yeast‐like cells, with sparse, rudimentary hyphal elements (Figure 3b). PCR analysis identified the species as *M. slooffiae*.

Figure 3Skin fungal cultures (Sabouraud Dextrose Chloramphenicol Agar with supplementation of olive oil, 37°C, 5 days). (a) Rare growth of smooth, cream‐colored colonies (gray rectangle). (b) Yeast microscopic observation (lactophenol cotton blue, ×1000).

**Figure:**
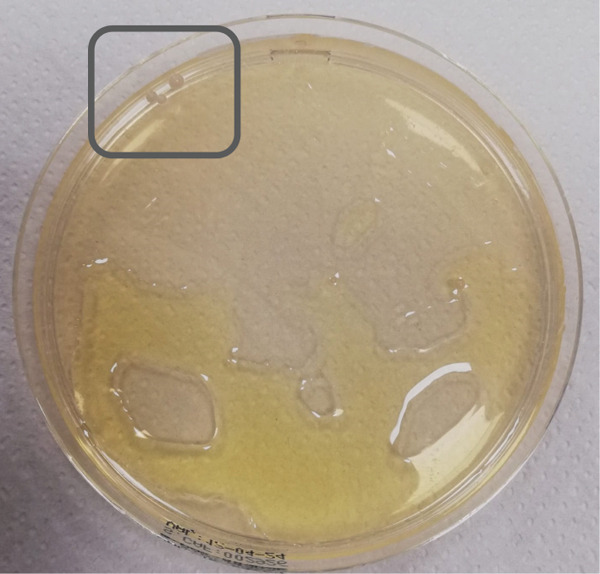
(a)

**Figure:**
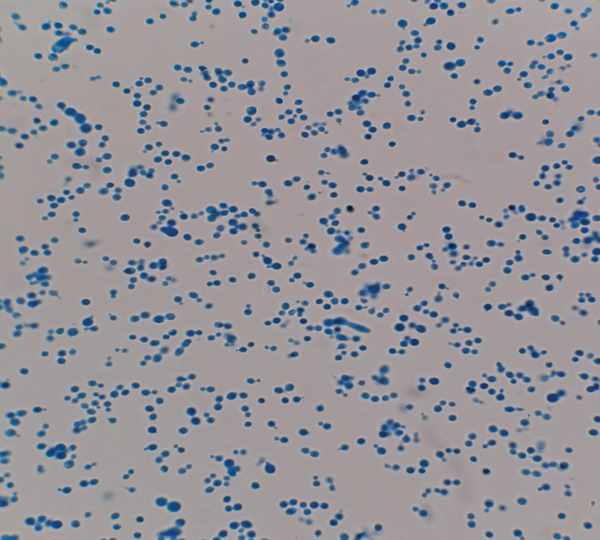
(b)

Two biopsy samples were taken from the bird′s gular pouch in a routine medical check‐up under general anesthesia: one from normal skin and one from a slightly hyperpigmented lesion. Histopathology of the hyperpigmented lesion revealed several foci of pericapillary infiltration of small lymphocytes (Figure 4a), which were not observed on the normal gular pouch sample (Figure 4b). In both samples, the stratum corneum exhibited variable thickness and consisted of keratin layers with low cohesion. Periodic acid–Schiff (PAS) staining revealed rare forms of yeasts between the layers of the stratum corneum of the hyperpigmented lesion (Figure 4c).

Figure 4Gular sac—histopathology. (a) Dermal perivascular lymphoid infiltration. PAS, ×100. (b) Normal structure with no perivascular cell infiltrates. PAS, ×100. (c) Skin surface. A few *Malassezia* sp. forms are present between the keratin layers (arrows). PAS, ×1000.

**Figure:**
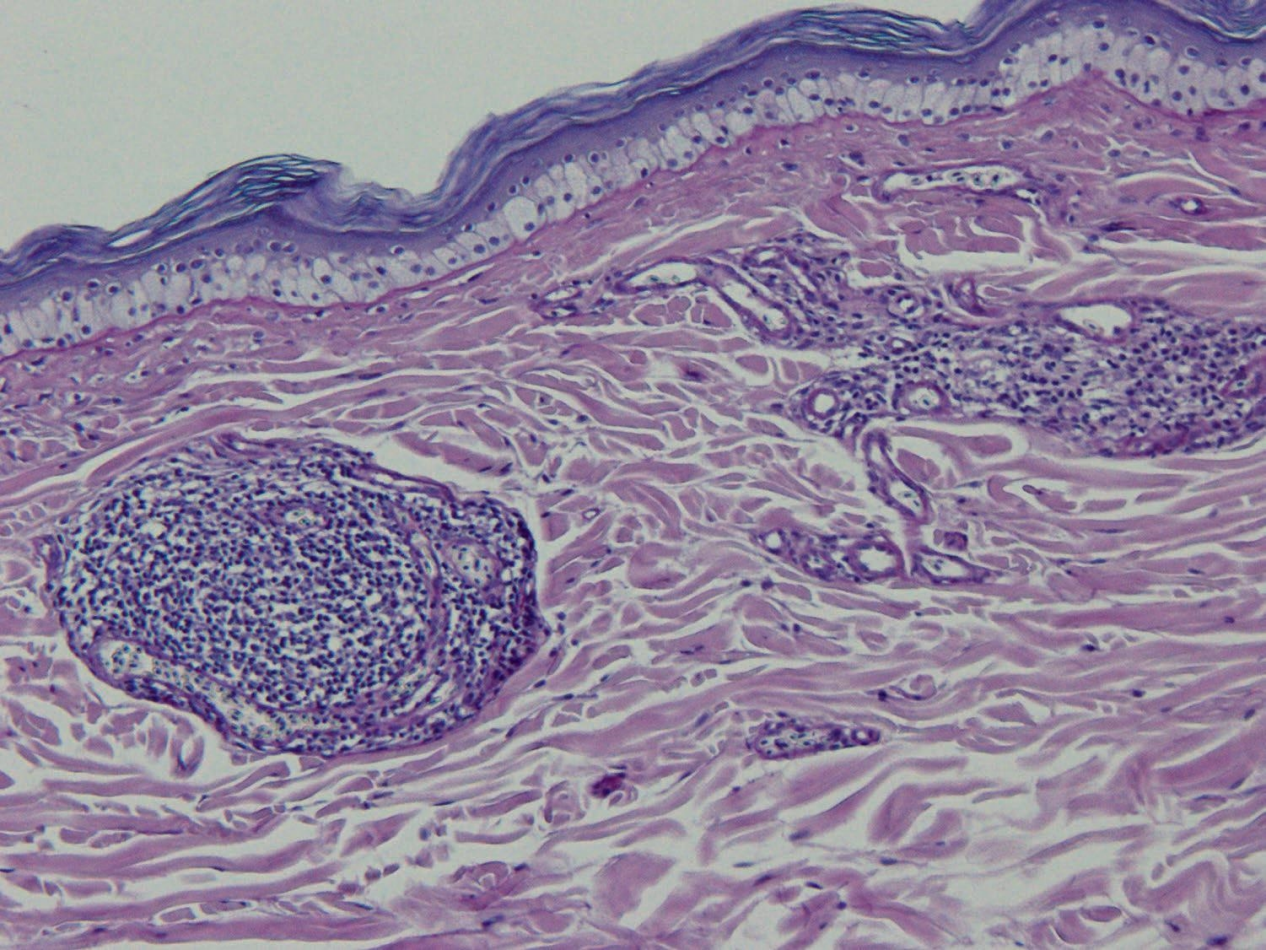
(a)

**Figure:**
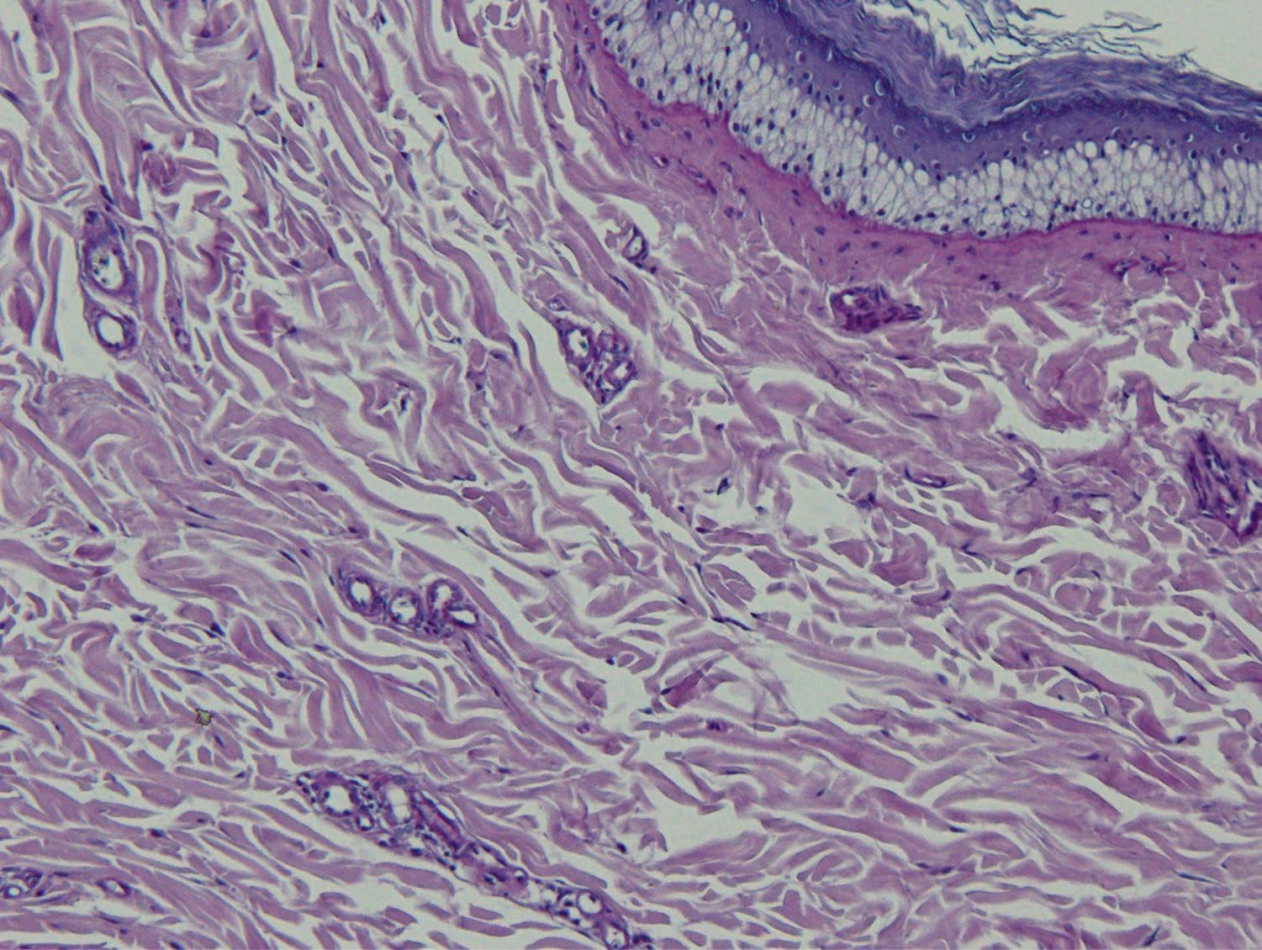
(b)

**Figure:**
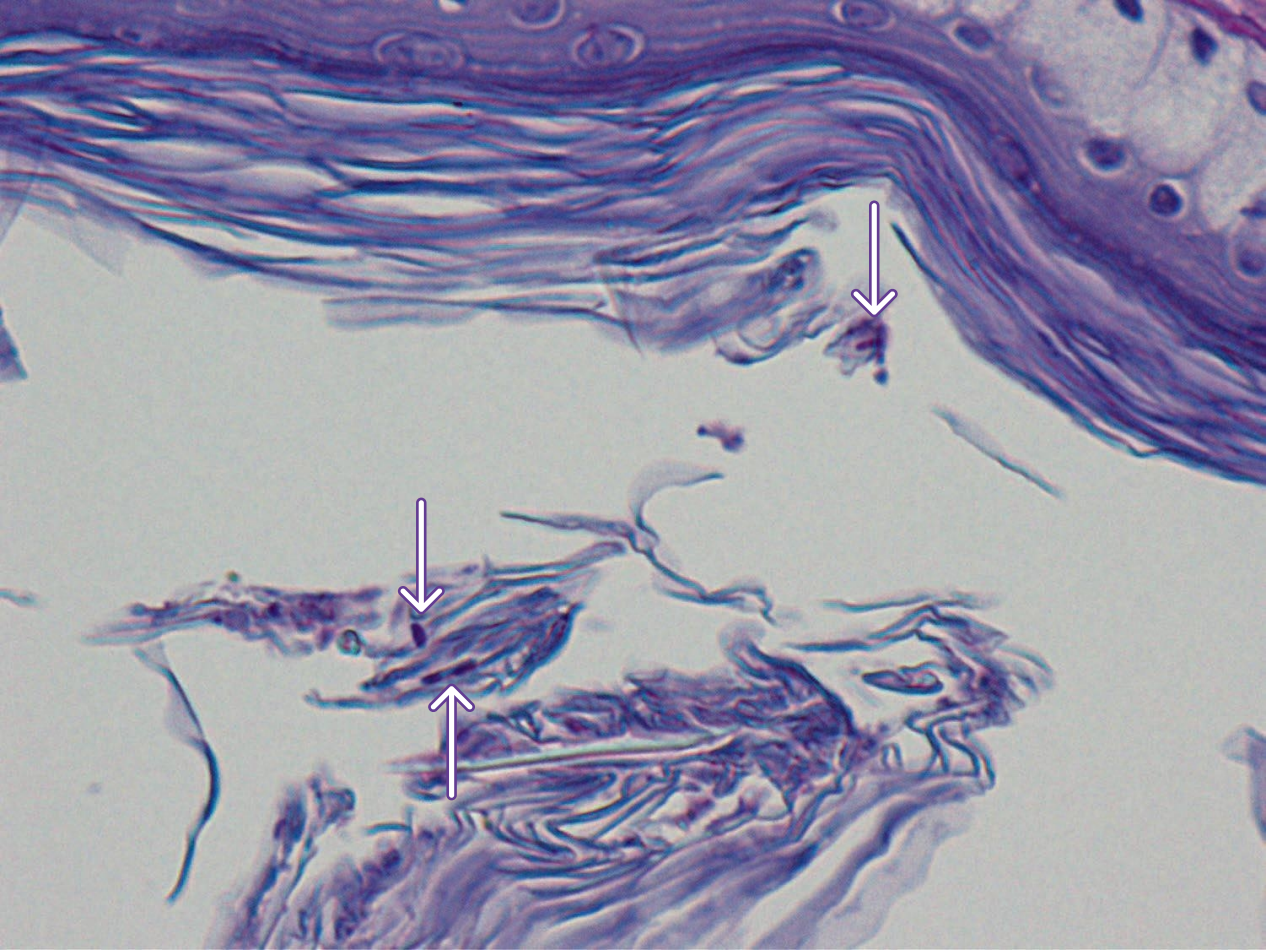
(c)

Notably, the lesions were self‐limiting and almost entirely resolved after 4 months without any therapeutic intervention (Day 131). However, on Day 309, several hyperpigmented macules reappeared in the gular pouch, similar to those previously described. This clinical recurrence was accompanied by an increased yeast count in the cytological examination (20 yeasts/OIF).

## 3. Discussion

The color of the gular pouch in birds can vary due to several physiological factors, such as testosterone levels, age, and female visitation status [10]. In this hornbill, however, there was no overall change in the gular pouch′s normal red coloration. Instead, only localized, roundish, dark‐red macules were observed, suggesting a nonphysiological process that had not been previously documented in the bird′s medical history.

Research on the avian skin microbiota remains limited, with scarce data on the species and number of yeasts present on the skin of healthy birds [6]. Cutaneous slides from the macules observed in this hornbill revealed more than 100 budding yeasts/OIF, a finding that in other species is associated with a local microbiota imbalance. In companion animals, *Malassezia* yeasts are rarely observed in cytological samples from healthy skin; however, the distinction between normal and abnormal numbers of *Malassezia* spp. is also not straightforward, as yeast counts may vary with climate conditions, body site, breed, and other individual characteristics [11]. As a general guideline, it is considered likely abnormal if more than one to two yeasts/OIF are observed in skin samples, particularly when accompanied by clinical signs such as erythema or pruritus [11].

Other macroscopically healthy skin areas of the southern ground hornbill, such as the chest and periocular skin, showed zero and up to eight yeasts/OIF, respectively. As described in mammals, these findings underscore the possibility of *Malassezia* spp. being part of the physiological avian skin microbiome, which can act as opportunistic pathogens [3]. Furthermore, though clear cytological improvement (Day 62: zero to one yeast/OIF) happened several weeks before the near clinical resolution of the hyperpigmented lesions (Day 131), the reappearance of the macules on Day 309 coincided with an increased number of *Malassezia* sp. forms (> 20 yeasts/OIF) in the follow‐up cytological evaluation.

In humans, several *Malassezia* species are known etiologic agents of pityriasis versicolor, atopic dermatitis, folliculitis, and seborrheic dermatitis [12]. Pityriasis (tinea) versicolor is a superficial fungal skin infection characterized by hyperpigmented or hypopigmented macules and finely scaled plaques, with a high rate of recurrence [4]. Diagnosis is usually made clinically; however, in individual cases, Wood′s lamp examination or microscopic examination of fungal elements is warranted [4]. Pityriasis versicolor has usually been associated with perivascular lymphocytic infiltration and hyperkeratosis [12]. In birds, the most common lesions associated with malasseziosis include hyperkeratosis with the presence of yeast forms in the stratum corneum, epidermal hyperplasia, and lymphoplasmocytic perivascular dermatitis, as described in the present case [7, 13]. The presence of *Malassezia* sp. exclusively on the hyperpigmented lesions on the gular sac of the hornbill suggests that these microorganisms may be involved in the pathophysiology of the perivascular inflammation and skin depigmentation of this hornbill.

In humans, the colonization of *Malassezia* spp. may be influenced by host features (e.g., genetics, immune response, body secretion, skin occlusion, and microbiota) and environmental parameters [4]. In companion animals, the pathogenic role of *Malassezia* spp. seems to be related to the host immune system as well as to yeast virulence factors [4]. Particular conditions that may predispose to *Malassezia* spp. overgrowth in these animals include atopic or seborrheic dermatitis, parasitic infestation, diabetes mellitus, viral infections, and long‐term antibiotic use associated with glucocorticoid treatment [4]. Although zoonotic transmission has been described for *M. pachydermatis*, there is currently no confirmed evidence of zoonotic transmission of *M. slooffiae* [14]. Though no risk factors can be directly linked to the localized *M. slooffiae* overgrowth in this hornbill, the pathogenic potential of this yeast may have been triggered by imbalances in the hornbill′s skin microenvironment and immune system. The medical history of this bird was overall unremarkable, with no previous history of acute or chronic illnesses, use of antibiotics, or immunosuppressive medications. Moreover, factors such as overcrowding or poor hygiene of the outdoor enclosure were dismissed, as this animal did not live with other birds, and the habitat was thoroughly cleaned. Other habitat and environmental conditions may have played a role in the development of the lesions, as they dissipated throughout late spring and summer, associated with a warmer but less humid environment, whereas the initial appearance and the recurrence of the lesions were observed in a colder but more humid season.

Even though changes in skin coloration are observed in humans with pityriasis versicolor, these have not been described in birds. This case offers further insight into the role of *Malassezia* spp. as a potential component of the normal microbiota of the avian skin, as well as its role in avian dermatologic conditions. Broader studies on the southern ground hornbill microbiome, as well as on normal skin cytology and histopathology characteristics, may be crucial for effective medical preventive and reactive care, as well as conservation strategies.

## Funding

No funding was received for this manuscript.

## Conflicts of Interest

The authors declare no conflicts of interest.

## Data Availability

The data that support the findings of this study are available from the corresponding author upon reasonable request.
